# Long-Read Metagenomics Profiling for Identification of Key Microorganisms Affected by Heavy Metals at Technogenic Zones

**DOI:** 10.3390/microorganisms14010196

**Published:** 2026-01-15

**Authors:** Iskander Isgandarov, Zhanar Abilda, Rakhim Kanat, Dias Daurov, Zagipa Sapakhova, Ainash Daurova, Kabyl Zhambakin, Dmitriy Volkov, Abylay Begaly, Malika Shamekova

**Affiliations:** 1 Institute of Plant Biology and Biotechnology, Almaty 050040, Kazakhstan; i.isgandarov@ipbb.kz (I.I.); z.abilda@ipbb.kz (Z.A.); r.kanat@ipbb.kz (R.K.); d.daurov@ipbb.kz (D.D.); z.sapakhova@ipbb.kz (Z.S.); a.daurova@ipbb.kz (A.D.); k.zhambakin@ipbb.kz (K.Z.); d.volkov@ipbb.kz (D.V.); a.begaly@ipbb.kz (A.B.); 2 Tanir Research Laboratory, 75B Al-Farabi Avenue, Almaty 050060, Kazakhstan

**Keywords:** heavy metal contamination, metagenomics, long-read sequencing, whole metagenome sequencing, soil microbial community

## Abstract

Heavy metal pollution poses a serious threat to soil ecosystems worldwide, as long-term exposure can alter microbial community functioning and reduce overall ecosystem resilience. This study investigated the impact of heavy metal contamination in technogenic industrial areas of the East Kazakhstan Region on soil microbial communities. Soil samples were collected for chemical and metagenomic analyses. Concentrations of Zn, Pb, Cu, and Cd were quantified by flame atomic absorption spectrometry (FAAS). Using long-read whole-metagenome nanopore sequencing, we conducted strain-level profiling of soils with different levels of metal contamination. This approach provided high-resolution taxonomic data, enabling detailed characterization of microbial community structure. Heavy metal exposure did not significantly reduce microbial diversity or richness but influences the quality of community composition. Metal-resistant taxa dominated contaminated soils. Overall, the results highlight the value of long-read sequencing for resolving strain-level responses to environmental contamination.

## 1. Introduction

Heavy metal (HM) contamination is a significant global issue, particularly in developing countries. The northern part of the East Kazakhstan Region has long been one of the major industrial centers of Kazakhstan. The mining and processing of non-ferrous metals have been carried out in the study area for the last 75–80 years. Heavy metals are hazardous for ecosystems and can affect human health directly or through food-chain bioaccumulation [1,2]. Chronic environmental exposure to heavy metals can cause direct and indirect effects on the human health [3,4,5]. The high toxicity of heavy metals has a complex effect on soil quality, binding with its mineral and organic colloids [6]. Because of their toxicity, they are the main cause of soil contamination [7]. Once heavy metals enter the soil, they chemically bind not only with all soil components, but also with plants (rhizosphere accumulation) and local microbial populations [8].

Microorganisms play crucial roles in soil ecosystems, maintaining soil health, supporting environmental sustainability, and mediating soil carbon cycling [9]. Microorganisms enhance plant growth by promotion of root growth, modulation of plant hormones by enhancing nutrient bioavailability, and, finally, helps to reduce stress from environmental factors including heavy metal pollution [10,11]. However, the tolerance of microorganisms varies across communities in relation to the concentration fluctuations of HMs. Depending on their chemical form and concentration, HMs damage microorganisms through production of reactive oxygen species (ROS), leading to oxidative stress, disruption of enzymatic functions, causing disbalance of metabolic processes, and direct damage to the cell membrane. Zinc and copper are essential metals that participate in biochemical processes necessary for life [12]. However, their high concentrations in biomass have a negative effect on the viability of microorganisms [13]. For instance, elevated Cu concentrations can impair enzymatic functions and disrupt cellular metabolic processes, whereas excessive Zn levels may decrease biomass accumulation and exert phytotoxic effects. Non-essential metal Pb causes enzymatic disturbances, water disbalance, and anomalies in metabolic functions [1,14]. Cd inhibits secretion of extracellular enzymes by soil microorganisms [15]. It is crucial to monitor how the qualitative and quantitative composition of the microbiome responds to HM concentrations. The sensitivity of microbial communities to fluctuations in the pool of freely available HM ions in soil reflects the actual level of HM bioavailability to plants [16].

Several studies have identified the effects of heavy metals on soils in technogenic zones [17,18]. For instance, Woszczyk et al. reported increased concentrations of cadmium (Cd), copper (Cu), zinc (Zn), and lead (Pb) in soils from Ust-Kamenogorsk in the East Kazakhstan region. In these soils, the enhanced bioavailability of these metals was noted, which could be potentially hazardous for the local microbiota [19].

Understanding how soil microbial communities respond to this complex contamination landscape requires sensitive, unbiased profiling methods. Next-generation sequencing (NGS) methods and metagenomics are powerful tools for studying biodiversity of ecosystems, as they capture comprehensive profiles of microbial communities without cultivation. Soil metagenomics can reveal microbial diversity patterns and identify potential pathways for soil remediation [20,21]. Multiple studies explored the effects of HMs on microbial diversity and community composition [22,23,24]. Several studies identified Proteobacteria and *Actinobacteria* as dominant soil phyla [24,25,26]. For instance, Su et al. demonstrated that Cd concentrations significantly affect microbial community composition [27]. Furthermore, long-term heavy metal exposure induces metal tolerance mechanisms that are essential for survival of microorganisms and maintenance of soils’ health in extremely polluted environments [28]. The study of Huang et al. documented significant shifts in microbial communities exposed to chromium (Cr), copper (Cu), nickel (Ni), and zinc (Zn) [29]. Several studies have identified metal tolerance in Proteobacteria, *Actinobacteriota*, and *Firmicutes* [8,30].

However, many of these studies are based on amplicon-based sequencing and short-read sequencing approaches [25,27,31,32]. These methods could demonstrate lower resolution and accuracy of sequencing compared to whole-metagenome sequencing combined with long-read-based methods [33,34]. Whole-metagenome sequencing captures all genetic material present in a complex microbial community without targeting or amplifying any particular marker genes in advance [35]. Long-read sequencing provides superior resolution at the genus and species level compared with short-read sequencing [36]. In the present study, long-read-based whole-metagenome sequencing was applied to identify key microorganisms at different taxonomic levels across all domains that are affected by heavy metals and to assess effects on microbial diversity and relative abundance. Specific objectives were to apply long-read metagenomics for high strain-level taxonomic resolution of soil microbial communities and to evaluate the effects of different concentration of heavy metals on soil microbial community composition across phylum, order, genus, and strain levels.

## 2. Materials and Methods

### 2.1. Study Area, Sample Collection and Soil Analysis

#### 2.1.1. Study Area

**Figure 1:**
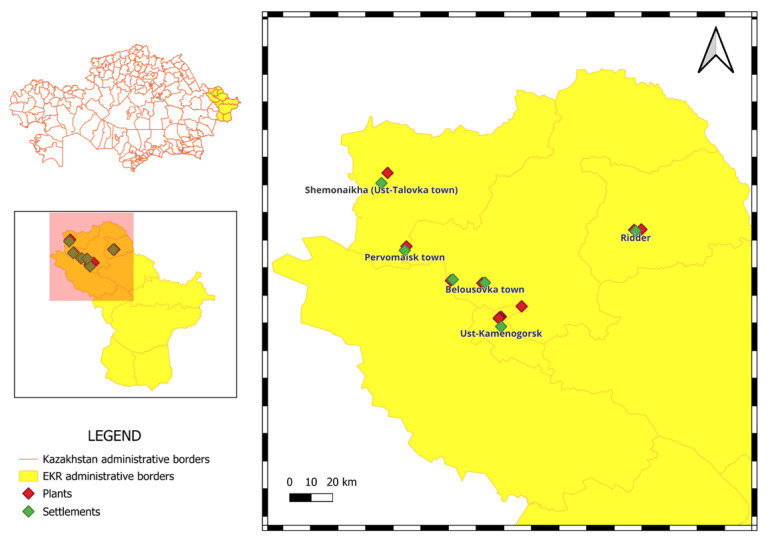
Area of study with settlements and plants.

The study area is located in the East Kazakhstan Region, including the cities of Ust-Kamenogorsk (49.97° N, 82.60° E), Ridder (50.35° N, 83.35° E), Shemonaikha (50.57° N, 81.86° E), Belousovka (50.13° N, 82.53° E), Glubokoe (50.13° N, 82.21° E), and Pervomaisk (50.26° N, 81.99° E). The area under study has long been an industrial center, with non-ferrous metal-processing plants located in its north and north-western parts since the 1940s. Currently, most of the industrial facilities are active and continuing its processing (Figure 1). Soil samples were collected near copper, lead, and other metal-processing plants. The selection was carried out in eight directions according to the wind rose at distances ranging from 250 to 3200 m from the boundaries of industrial facilities. The climate of East Kazakhstan Region is characterized by great diversity. The sharp continentality of the desert and semi-desert areas of the region is significantly smoothed out in the mountainous and foothill areas. The warm period with an average daily temperature above 0° in the north-east of the region lasts less than 200 days (mountainous and foothill areas), and in the south of the region from 200 to 230 days (steppe, semi-desert, and desert areas). The distribution of annual precipitation is uneven. In the north-east of the region, it is 400–650 mm (mountainous and foothill areas), with the lowest precipitation falling in the intermountain basins—less than 200 mm per year. Average wind speeds are 2–5 m/s. However, in some areas of the region, strong winds (15 m/s and above) are not uncommon (https://www.kazhydromet.kz/).

#### 2.1.2. Soil Sampling

Samples were collected at industrial areas, near settlements, and 200 to 4000 m from destination zones. A total of 33 soil samples were strategically selected based on their heavy metal (HM) concentrations to facilitate a more precise assessment of the relationship between metals and microorganisms. The study did not designate specific control samples. Soils were sampled for chemical and metagenomic analyses. Samples were collected by removing the top layer of soil (0 to 5 cm) and collecting approximately 500 g of soil at a depth of 5 to 10 cm. To determine HM content, the soil was air-dried at room temperature for one week and then sieved through a 100-mesh sieve to remove stones and visible plant fragments. Part of the soil was immediately frozen in liquid nitrogen and stored at −80 °C for subsequent molecular analysis.

#### 2.1.3. Atomic Absorption Spectrometry

Concentrations of Zn, Pb, Cu, and Cd were determined by flame atomic absorption spectrometry (FAAS) using an AA 240 instrument (Agilent, Santa Clara, CA, USA) in accordance with ISO 11047 [37]. AAS is a sensitive method with a wide range of metal detection.

The limits of detection (LOD) and quantification (LOQ) were determined as three and ten times the standard deviation of blank measurements, respectively. The LOD/LOQ values were as follows: Zn—0.001/0.03 mg L^−1^, Cu—0.001/0.03 mg L^−1^, Pb—0.001/0.03 mg L^−1^, Cd—0.001/0.03 mg L^−1^. The measured heavy metal concentrations were compared with the maximum permissible concentrations (MPCs) established in Kazakhstan (https://adilet.zan.kz/rus/docs/V1500011755, accessed on 5 November 2025) to assess potential environmental risk, as well as with guideline values proposed by the Food and Agriculture Organization (FAO) (https://faolex.fao.org/docs/pdf/est97999E.pdf, accessed on 5 November 2025).

### 2.2. DNA Extraction, Library Construction, and Sequencing

DNA from the selected 33 samples was extracted using the DNeasy Power Soil Kit (Qiagen, Hilden, Germany) according to the manufacturer’s instructions and purified with AMPure XP Beads (Beckman Coulter, Brea, CA, USA) to bring the DNA to the required specifications. The A260/280 and A260/230 ratios were checked on NanoDrop 2000 (Thermo Fisher Scientific, Waltham, MA, USA). Libraries were prepared using the Native Barcoding Kit 24 V14 (SQK-NBD114.24, Oxford Nanopore Technologies, Oxford, UK) following the manufacturer’s protocol, including DNA repair/end-prep, native barcode ligation, and adapter ligation with AMPure XP bead purification. For multiplexing, samples were pooled equimassively. Final libraries were loaded onto R10.4.1 flow cells and sequenced on the PromethION 2 Solo (P2 Solo) device using MinKNOW software (version 25.05.14), (Oxford Nanopore Technologies, Oxford, UK). Basecalling and demultiplexing were performed using MinKNOW with the corresponding kit configuration.

### 2.3. Metagenomic Data Preprocessing and Analysis

After sequencing, raw reads were processed using the NanoPack suite. Read quality filtering was performed with NanoFilt, and NanoPlot used to visualize read length and quality distributions. Reads were filtered using a minimum quality score threshold of Q15 and minimal read length of 300 bp. Taxonomic mapping and classification of filtered reads were performed using MetaMaps with default parameters, which achieves strain-level resolution through a reference-based long-read mapping approach against the complete RefSeq database. All statistical analyses were conducted in R (version 4.5.1).

Continuous variables were compared across groups using the Kruskal–Wallis test, and Spearman correlations were performed to assess relationships in the microbial community, with significance level *p* < 0.05. Bray–Curtis distance matrices were constructed to assess differences in metagenomic composition among groups, and the differences between sampling locations were evaluated using permutational multivariate analysis of variance (PERMANOVA) with 999 permutations, implemented in the adonis2 function in vegan package (version 2.7-2). Alpha diversity (OTU, Chao1, Shannon, and Simpson diversity indexes), Hellinger Transformation, non-metric multidimensional scaling (NMDS), and Redundancy analysis (RDA) were applied to relate community composition to heavy metal concentrations, and the significance of the global RDA model was assessed with 999 permutations using the anova.cca function in vegan package (version 2.7-2). Mantel tests based on Spearman correlation were performed with the linkET (version 0.1.0) package to evaluate the relationship between community dissimilarity and environmental distance matrices.

## 3. Results

### 3.1. Soil Heavy Metal Composition and Sampling Zones

The localization of sampling sites, including their geographic coordinates and heavy metal concentrations, is presented in (Table 1). In total, 33 samples were collected from seven technogenic zones of six settlements. The concentrations of all measured heavy metals differed significantly among technogenic zones (*p* < 0.05). For reference, the maximum permissible concentrations (MPCs) established in Kazakhstan and the guideline values proposed by the FAO are 0.5 and 1 mg/kg for cadmium, 33.0 and 100 mg/kg for copper, 32.0 and 50 mg/kg for lead, and 23.0 and 200 mg/kg for zinc, respectively.

### 3.2. Taxonomic Composition

**Figure 2:**
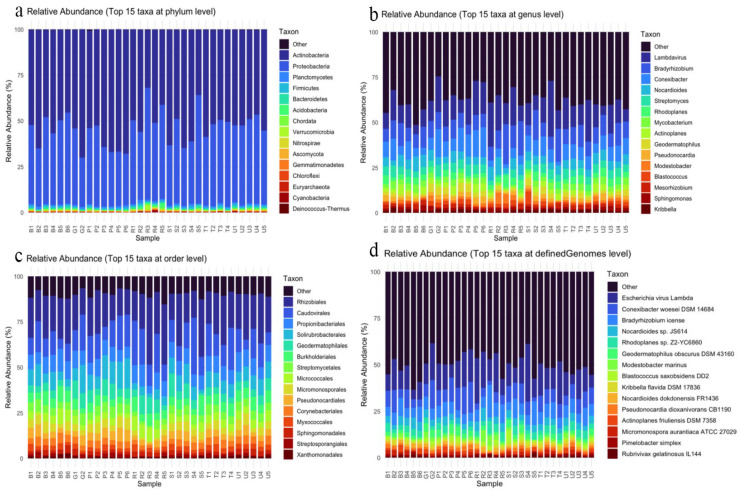
Stacked bar charts demonstrating relative abundance of top taxa (**a**) at phylum level; (**b**) at order level; (**c**) at genus level; (**d**) at the strain level.

Taxonomic annotation and classification were performed using Metamaps. Metamaps classified microorganisms from four domains: Archaea (0.237%), Eukaryota (0.655%), Viruses (9.20%), and Bacteria (89.9%). Classification resolved reads down to the strain level. Top 15 most abundant taxa from phylum, order, genus, and strain levels are shown in (Figure 2). At the phylum level (Figure 2a) most abundant microorganisms were *Proteobacteria* and *Actinobacteria*. At the order level (Figure 2b), they are *Rhizobiales*, *Caudovirales*, and *Propionibacterialis*. At the genus level (Figure 2c), *lambdavirus*, *Bradyrhiszobium*, *Conexibacter*, and *Nocardioides* predominated. The most abundant strains (Figure 2d) were *Escherichia Virus lambda*, *Conexibacter woesei* DSM 14684, *Bradyrhizobium icense*, and *Nocardioides* sp. JS614.

### 3.3. Alpha Diversity and Relationships to Metals

The alpha diversity indices calculated from the classified data were applied to evaluate the richness and diversity of microbial communities across all samples (Table 2). The Mean OTU was 1707 (±251), while the mean Chao1, Shannon, and Simpson indices were 2050 (±315.1), 4.761 (±0.141) and 0.9682 (±0.0015), respectively. The highest OTU and Chao1 values were observed in sample P3 (2115 and 2440, respectively), whereas the lowest OTU and Chao1 were indicated in sample S4 and their values were 1161 and 1290, respectively. There were no statistically significant differences in alpha diversity indices among the different sampling locations (*p* > 0.05).

**Figure 3:**
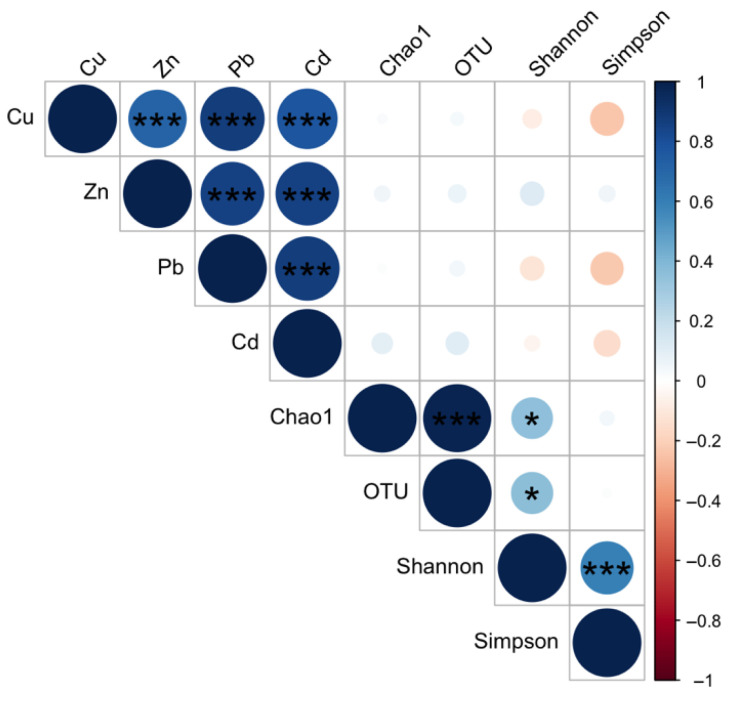
Spearman correlation test between alpha diversity indexed and heavy metals. Asterisks indicate levels of statistical significance: *** *p* < 0.001, and * *p* < 0.05.

To assess the relationship between alpha diversity and heavy metal concentration, Spearman correlation was applied (Figure 3). No statistically significant correlations were detected between heavy metals and the alpha diversity indices.

### 3.4. Relationship Between Microbial Communities and Heavy Metals

**Figure 4:**
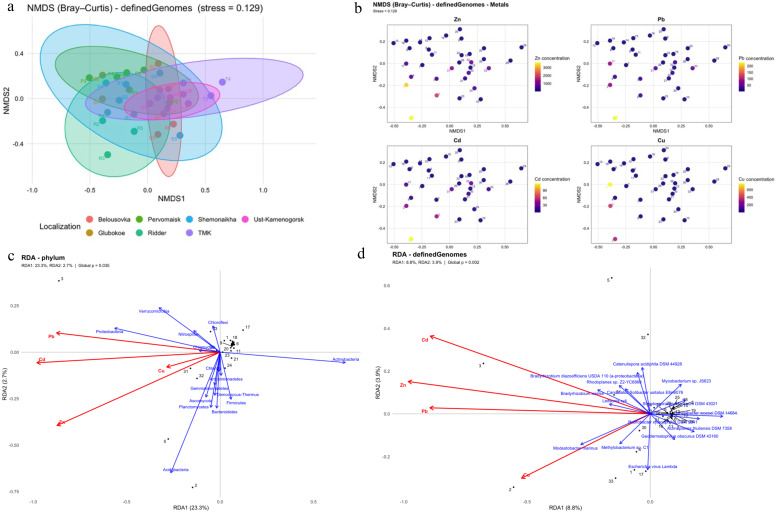
Influence of heavy metals on soil communities: (**a**) NMDS plot visualizing different sampling areas, (**b**) NMDS plot demonstrating distribution of heavy metal concentrations across sampling sites, (**c**) RDA plot at phylum level, (**d**) RDA plot at the strain level.

At the strain level, the ADONIS test indicated significant differences in microbial community composition among different sampling areas (F = 1.46, *p* = 0.036). Non-metric multidimensional scaling (NMDS) ordination (Figure 4a) was applied to reduce the high-dimensional data. Stress level < 0.2 provides a good representation of the dissimilarity structure in reduced dimensions. An additional NMDS ordination colored by the concentrations of four heavy metals (Figure 4b) was utilized to visualize how community composition varied along the Zn, Cu, Cd, and Pb gradients. A combined ADONIS model revealed that sampling location and metal concentrations (Pb, Zn, Cu, Cd), together with their interactions, significantly influenced the microbial community composition (F = 1.36, *p* = 0.02), explaining approximately 45% of the total variation.

Redundancy analysis (RDA) at the phylum level identified relationships between heavy metal concentrations and the 15 most abundant phyla in this dataset (Figure 4c). In this model, heavy metals explained 26% (RDA1 explained 23.3% and RDA2 explained 2.7%) of the variance in community structure. Permutation Test for Redundancy Analysis confirmed a significant overall effect of Zn, Cu, Cd, and Pb on the phylum-level community (F = 2.7086, *p* = 0, 022). In the ordination space, *Proteobacteria*, *Verrucomicrobia*, *Nitrospirae*, and *Chlamydae* demonstrated strong positive associations with the Pb and Cd vectors. In contrast, *Actinobacteria* demonstrated strong negative associations with all heavy metal vectors. At the strain level, RDA revealed associations between heavy metal concentrations and the 15 most abundant strains (Figure 4d). Here, heavy metals explained 12,7% (RDA1 explained 8.8% and RDA2 explained 3.9%) of the variance in community composition. Permutation Test for Redundancy Analysis again indicated significant effects of Zn, Cu, Cd, and Pb on the strain-level microbial community (F = 1.5145, *p* = 0.001). *Bradyrhizobium icense*, *Leifsonia xyli*, *Candidatus Solibacter usitatus* Ellin6076, and *Rhodoplanes sp.* Z2-YC6860 were strongly positively associated with the Cd, Zn, and Pb vectors, whereas *Modestobacter marinus* and *Methylobacterium* sp. C1 were positively associated with the Cu vector. Conversely, *Conexibacter woesei* DSM 14684, *Rubrobacter xylanophilus* DSM 9941, and *Streptosporangium roseum* DSM 43021 showed negative associations with all four heavy metals.

**Figure 5:**
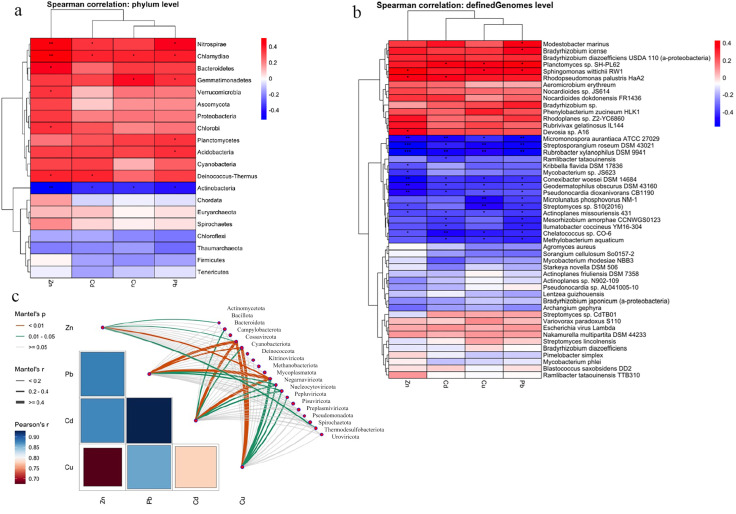
Correlations between heavy metals and soil communities. (**a**) Spearman correlation at phylum level; (**b**) Spearman correlation at the strain level; (**c**) Mantel test between heavy metal and community matrices. Asterisks indicate levels of statistical significance: * *p* < 0.05, ** *p* < 0.01, and *** *p* < 0.001.

Spearman correlation analysis revealed that microbial taxa exhibited distinct responses to varying heavy metal concentrations. At the phylum level, two main clusters were observed (Figure 5a). Cluster 1 showed significant positive correlations with heavy metals, while cluster 2 showed weak or negative correlations, particularly within the phylum *Actinobacteria*. A similar pattern was observed at the strain level (Figure 5b). Cluster 1 strains were significantly positively correlated with metal concentrations, while cluster 2 strains were negatively correlated with one or more metals.

A Mantel test was applied to assess correlations between overall soil microbial community structure and heavy metal concentrations (Figure 5c). Strains belonging to the phyla *Campylobacterota* and *Mycoplasmatota* were significantly correlated with all heavy metals. Members of *Cossaviricota*, *Negarnaviricota*, and *Nucleocytoviricota* were associated with each of Pb, Cd, and Cu. Strains from *Actinobacteria*, *Bacillota*, *Peploviricota*, and *Thermodesulfobacteriota* phyla were associated with any of the heavy metals.

## 4. Discussion

This study shows that long-read whole-metagenome sequencing can identify strain-level responses of soil microbial communities to chronic heavy metal contamination across multiple technogenic zones. The results indicate that metals do not reduce overall diversity but strongly restructure community composition and selectively enrich or suppress specific phyla and strains with clear implications for bioremediation.

By applying long-read nanopore sequencing, the metagenomes were resolved down to the strain level, allowing direct comparison of strain-specific metal responses that are typically obscured in 16S or short-read studies. Taxonomic profiling identified four domains across all samples: Bacteria (89.9%), Viruses (9.20%), Eukaryota (0.655%), and Archaea (0.237%). The predominance of *Proteobacteria*, *Acidobacteria*, and *Actinobacteria* at the phylum level is consistent with previous soil metagenomics studies [38,39]. These patterns may be related to the well-documented heavy metal tolerance reported for *Proteobacteria* and *Acidobacteria* [40,41]. At the order level, we identified *Rhizobiales*, which are considered important microorganisms for the removal of Cd from long-term contaminated soils [42]. Some members of the order *Propionibacteriales* have been reported as effective degraders of several pollutants [43]. At the genus level, *Nocardioides* and *Bradyrhizobium* play established roles in bioremediation and have been identified as tolerant to several pollutants, including heavy metals [43,44,45]. *Conexibacter* has also been described as a heavy metal-resistant genus [46,47]. In this study, only one dominant viral taxon was detected. It is *Escherichia Virus lambda*, belonging to the order *Caudovirales* and the genus *Lambda Virus. Caudovirales* is the most prevalent viral order in soils and is involved in regulation of bacterial populations through lysis [48,49]. Members of *Caudovirales* participate in horizontal gene transfer, including the dissemination of antibiotic resistance genes [48]. *Escherichia virus lambda* is a temperate bacteriophage that infects *Escherichia coli* [50]. Based on its dominance in dataset, *E. virus lambda* may act as a key regulator of *E. coli* populations in these technogenic soils through lysogenic–lytic dynamics. Among bacterial strains, *Conexibacter woesei* DSM 14684 belonging to the *Conexibacter*, was detected as one of the most abundant taxa. Previous studies have reported that the abundance of *Conexibacter* is associated with structured and stable soils, where these bacteria grow within protected microenvironments [51]. Results contrast with these observations. *C. woesei* DSM 14684 was abundant under conditions of pronounced heavy metal pollution, suggesting that this strain can persist in stressed technogenic soils or that specific microhabitats still provide protection despite overall contamination. *Bradyrhizobium icense*, a nitrogen-fixing species within the genus *Bradyrhizobium*, was also abundant. The genus *Bradyrhizobium* has recognized bioremediation potential and exhibits resistance to multiple heavy metals [52]. Taken together, these findings suggest that soils contaminated with heavy metals in technogenic zones are dominated by metal-resistant microorganisms that are less influenced by heavy metals. In addition, communities contain nitrogen-fixing microorganisms essential for bioremediation process and maintenance of soil health.

This study shows that highly diverse microbial communities were identified across all technogenic zones. Despite notable variation in heavy metal levels, no significant correlations were observed between heavy metal concentrations and alpha diversity indices. These findings contrast with many previous studies reporting that heavy metal pollution significantly reduces the diversity and richness of soil communities [24,53]. These findings support that, under chronic heavy metal exposure, soil microorganisms can maintain community-level diversity and function by developing metal tolerance mechanisms and adapting to persistent stress conditions [54]. Tipayno et al. showed that heavy metal pollution primarily affects rare taxa and that adaptation of bacterial communities to heavy metal contamination is driven predominantly by the replacement process, rather than a richness loss [55].

ADONIS test on strain-level data showed highly significant effects of sampling location, metal concentrations, and their interactions (F = 1.36, *p* = 0.02), indicating that heavy metals are important drivers of variation in microbial community structure and that their influence differs among technogenic zones. These results agree with other studies that identified heavy metals as key determinants of soil microbial community composition [56,57]. At the phylum level, RDA revealed that heavy metals collectively explained 26% of variance (RDA1: 23.3%, RDA2: 2.7%), with significant results for the permutation test (F = 2.71 *p* = 0.022). Correlation analyses and Mantel tests supported these findings, confirming a significant influence of heavy metals on microbial communities. The RDA biplot identified distinct response patterns among major phyla. *Proteobacteria*, *Verrucomicrobia*, *Nitrospirae*, and *Chlamydiae* were strongly positively associated with Pb and Cd vectors, suggesting that these taxa are either directly enriched by or specifically tolerant to these metals. Correlations and Mantel’s test supplement these results. This finding is consistent with the literature confirming metal resistance of *Proteobacteria* and other microorganisms [40,58]. Zou et al., in their study, indicated that *Nitrospirae* enriched under Zn and Pb pollution [59]. In striking contrast, *Actinobacteria* demonstrated strong negative associations with all metal vectors, indicating suppression of this otherwise abundant phylum under metal stress. This pattern suggests that, although *Actinobacteria* are generally dominant in many soils, they may be particularly sensitive to the combined metal burden in studied technogenic zones, consistent with previous observations of *Actinobacteria* sensitivity to heavy metals [60]. One plausible explanation is that, although some *Actinobacteria* possess robust metal resistance mechanisms (biosorption, bioaccumulation, biotransformation), many soil-dwelling actinobacterial taxa lack comprehensive metal efflux systems compared with metal-specialist *Proteobacteria* [61,62]. At the strain level, the variance explained by heavy metals decreased to 12.7% (RDA1: 8.8%, RDA2: 3.9%, *p* = 0.001), indicating that metal stress is a dominant predictor at higher taxonomic resolution but becomes less deterministic as one considers individual strains, where microhabitat effects and strain-specific traits likely increase variability. Spearman correlation analysis further supported a strong influence of HMs at strain-level resolution, highlighting both positively and negatively associated taxa. Several strains of *Bradyrhizobium* showed positive correlations with heavy metals. As mentioned before, *Bradyrhizobium* is tolerant to pollutants. *Rhodoplanes* sp. Z2-YC6860 was associated with Cd, Zn, and Pb vectors. Rosa et al., in their study, suggested that environmental pressure can drive solute-binding proteins duplication and adaptation of such organisms under suitable environmental factors [63]. *Modestobacter marinus* showed a selective positive correlation and association with Cu. Jiang et al. identified the presence of copper-resistance-related genes and other metal-resistance determinants, which support adaptation to heavy metal-induced stress [64]. *Sphingomonas wittichii* RW1 showed significant correlations with Cu, Zn, and Pb, likely reflecting to metal resistance associated with metal-resistant genes and controlling metabolic processes [65,66]. Several studies have demonstrated the efficiency of *S. wittichii* RW1 in degrading various organic pollutants [67,68]. Hu et al., in their study, defined *Sphingomonas* as important bioremediation microorganism in contaminated soils [57]. In contrast, *Conexibacter woesei*, *Rubrobacter xylanophilus* DSM 9941, *Streptosporangium roseum* DSM 43021, *Ramlibacter tataouinensis*, and *Streptomyces* sp. S10 (2016) were significantly suppressed under influence of HMs. It is previously mentioned that *Conexibacter* genus is mainly presented in stable and structured soils and has several heavy metal resistance mechanisms, but in this study, heavy metals significantly reduces abundance of this taxon. Streptomyces sp. S10 (2016) are also suppressed by heavy metals despite *Streptomyces* being broadly recognized as a metal-tolerant genus [61,69]. Similarly, *Rubrobacter xylanophilus*, *Ramlibacter tataouinensis*, and *Streptosporangium roseum*—all deep-branching *Actinobacteria*—were suppressed by heavy metals.

One of the limitations of this study is absence of physiological parameters (pH, organic matter, CO_2_, H_2_O). Integrating these variables would better separate direct metal effects from indirect environmental drivers and improve explanatory power. The functional implications of the observed taxonomic patterns remain inferred rather than demonstrated. Future studies should consider specific genes and metabolic pathways, while additionally providing metagenomic assembly and functional annotation of microbial communities. Even with these constraints, the work demonstrates that long-read whole-metagenome sequencing, combined with multivariate ecological analyses, is a powerful strategy to disentangle taxon- and strain-specific responses to chronic heavy metal contamination in complex soil environments.

## 5. Conclusions

Microorganisms are essential components of soil ecosystems and play crucial roles in soil physiochemical processes. In this study, we applied long-read whole-metagenome sequencing to provide comprehensive, strain-level characterization of soil microbial community composition, richness, and diversity across technogenic zones with varying heavy metal contamination levels. This long-read metagenomic approach achieved strain-level taxonomic resolution review on soil microbial community, unlike many metagenomic studies, that determine community resolution primarily at phylum level. Importantly, this study did not identify statistically significant differences in microbial diversity and richness under qualitive and quantitative heavy metal exposure. However, we found substantial shifts in microbial community composition of soil. Metal-resistant microorganisms predominated in contaminated soils. Species belonging to the phyla *Proteobacteria* and *Acidobacteria* were enriched, whereas the relative abundance of *Actinobacteria* was reduced. Additionally, we identified species involved in bioremediation process that may potentially contribute to heavy metal removal from soils. These compositional shifts could influence the biochemical properties of soils, although further confirmation is needed. In future research, long-read-based studies should aim to recover complete microbial genomes and identify specific genes and pathways involved in microbial response to hazardous heavy metal exposure.

## Figures and Tables

**Table 1:** Heavy metal concentrations across different samples and sampling areas.

Sample	Localization	Zn (mg/kg)	Cu (mg/kg)	Cd (mg/kg)	Pb (mg/kg)	Coordinates (Longitude/Latitude)	
R1	Ridder	518	11.3	4.8	25.2	50.35228	83.51445
R2	Ridder	3276	392	39	82	50.35231	83.50495
R3	Ridder	3624	328	118	227	50.35086	83.48562
R4	Ridder	578	1.9	6.4	20.2	50.34166	83.49445
R5	Ridder	1935	8.6	48	29.4	50.36110	83.50329
S1	Shemonaikha	9.9	1.2	0.4	0.6	50.59161	81.88157
S2	Shemonaikha	3.8	1.4	0.6	1.9	50.58868	81.91599
S3	Shemonaikha	5.7	0.7	1.9	0.7	50.58402	81.87749
S4	Shemonaikha	64	3.2	2.2	2.3	50.54346	81.85400
S5	Shemonaikha	2.1	6	1	1.3	50.55759	81.84727
P1	Pervomaisk	81	1.5	0.6	1.5	50.27801	82.00498
P2	Pervomaisk	27.8	2	1.7	2.5	50.29331	82.01324
P3	Pervomaisk	7.1	0.9	1.5	1.7	50.27764	82.01912
P4	Pervomaisk	1.5	1.5	1.6	0.7	50.27325	82.02766
P5	Pervomaisk	15.9	1.7	2.4	1.6	50.26831	82.01295
P6	Pervomaisk	23.6	2.5	1.5	3.4	50.26299	82.07384
G1	Glubokoe	442	762	17	85	50.13480	82.31807
G2	Glubokoe	17.5	5.8	3.1	4.2	50.16155	82.30049
B1	Belousovka	12.4	1.9	1.1	0.9	50.13000	82.47115
B2	Belousovka	120	10.5	3.1	4.3	50.13005	82.48750
B3	Belousovka	224	0.9	2.4	1.5	50.12204	82.49545
B4	Belousovka	13	0.8	0.7	0.7	50.11832	82.50514
B5	Belousovka	139	3.8	2.8	2.7	50.12392	82.51152
B6	Belousovka	453	8.1	7.1	6	50.13008	82.51066
T1	TMK	39.2	1.6	2.4	1.9	50.02666	82.74874
T2	TMK	9	0.5	0.9	0.7	50.02682	82.77135
T3	TMK	26.1	1.1	1.1	1.4	50.03651	82.77000
T4	TMK	37.8	1.3	2.2	2.2	50.03692	82.76096
U1	Ust-Kamenogorsk	591	6.4	10.9	22.9	50.03403	82.73848
U2	Ust-Kamenogorsk	1255	12.9	28.8	42	50.00077	82.58354
U3	Ust-Kamenogorsk	862	5.9	27	19	49.98743	82.63815
U4	Ust-Kamenogorsk	818	24.1	13.1	45	49.97572	82.60204
U5	Ust-Kamenogorsk	52	1.9	1.8	2.2	49.98978	82.58381

**Table 2:** Alpha diversity indices (Shannon, Simpson, Chao1, OTU) at different samples.

Sample	Shannon	Simpson	Chao1	OTU
B1	4.95	0.969	2190	1834
B2	4.6	0.968	1570	1356
B3	4.88	0.969	2202	1796
B4	4.86	0.969	2120	1723
B5	4.99	0.968	2328	1968
B6	4.91	0.969	2124	1746
G1	4.83	0.969	2467	2047
G2	4.47	0.966	2013	1701
P1	4.83	0.97	2419	2009
P2	4.74	0.969	1675	1421
P3	4.77	0.967	2440	2115
P4	4.73	0.968	2138	1769
P5	4.53	0.967	1775	1528
P6	4.55	0.967	1712	1423
R1	4.64	0.968	1792	1484
R2	4.63	0.968	2265	1825
R3	4.61	0.963	2510	2042
R4	4.62	0.968	2061	1742
R5	4.86	0.967	2580	2163
S1	4.81	0.968	2108	1746
S2	4.77	0.967	2295	1905
S3	4.73	0.968	2333	1912
S4	4.45	0.964	1290	1161
S5	4.81	0.966	2135	1731
T1	4.76	0.969	1758	1456
T2	4.85	0.969	2165	1768
T3	4.83	0.968	1739	1405
T4	4.67	0.968	1439	1216
U1	4.9	0.97	2091	1726
U2	4.87	0.967	2126	1812
U3	4.87	0.97	2055	1694
U4	4.78	0.968	1978	1623
U5	4.93	0.968	1736	1498

## Data Availability

The raw data supporting the conclusions of this article will be made available by the authors on request.
